# Improving Appendicitis Prediction in Children Using the Paediatric Appendicitis Score (PAS): A Three-Year Retrospective Study

**DOI:** 10.7759/cureus.91019

**Published:** 2025-08-26

**Authors:** Mhd Anas Murad, Mohamed Elgaml, Mohamad Wahib Zhlawi, Janani Rajesh Kumar, Gamalaldeen Abdulaal, Bandipalyam V Praveen, Emma Gray

**Affiliations:** 1 General and Colorectal Surgery, Southend University Hospital, Mid and South Essex NHS Foundation Trust, Southend-on-Sea, GBR; 2 Trauma and Orthopaedics, Southend University Hospital, Mid and South Essex NHS Foundation Trust, Southend-on-Sea, GBR; 3 Cardiology, The Princess Alexandra Hospital NHS Trust, Harlow, GBR; 4 General and Colorectal Surgery, Anglia Ruskin University, Chelmsford, GBR; 5 General and Breast Surgery, Southend University Hospital, Mid and South Essex NHS Foundation Trust, Southend-on-Sea, GBR

**Keywords:** acute appendicitis, appendicitis score, diagnosis, negative appendicectomy, paediatric

## Abstract

Introduction

Acute appendicitis is a leading surgical emergency in children, yet diagnosis remains challenging. The Paediatric Appendicitis Score (PAS) was developed to standardise risk stratification using clinical and laboratory indicators. While its diagnostic accuracy has been validated in controlled settings, its real-world application remains variable. This paper aimed to evaluate the impact of implementing PAS into routine clinical assessment at an NHS district-general hospital. The objectives were to reduce negative appendicectomy rates and improve diagnostic accuracy.

Materials and methods

A two-cycle retrospective audit was conducted at Southend University Hospital. Cycle 1 (August 2021-August 2024) included 156 paediatric patients who underwent appendicectomy. In August 2024, an educational intervention was introduced. It involved departmental teaching and dissemination of a quick-reference guide aligned with the Getting It Right First Time (GIRFT) abdominal pain pathway. Cycle 2 (August 2024-March 2025) included 34 cases. Patients under 18 years undergoing appendicectomy were included. The data collected included demographics, PAS scores, and histological outcomes. Diagnostic performance of PAS was evaluated by plotting the PAS value against the final histological outcome, using sensitivity, specificity, predictive values, likelihood ratios, and area under the receiver operating characteristic curve (AUC-ROC).

Results

A total of 190 patients were analysed. The negative appendicectomy rate fell from 29.5% (Cycle 1) to 17.6% (Cycle 2) following the educational intervention, although this was not statistically significant (p=0.16). In the combined cohort, PAS ≥7 achieved 87% specificity and positive predictive value (PPV) of 0.89, but only 40% sensitivity. PAS ≥4 maximised sensitivity (96%) but reduced specificity (31%). Mean PAS was significantly higher in confirmed cases (6.18 vs 4.69; p < 0.001). The AUC-ROC was 0.73.

Discussion

Across the full cohort, PAS showed expected trade-offs: at a high threshold (≥7), the tool provided good specificity (87%) but reduced sensitivity (40%), whereas a low threshold (≥4) delivered high sensitivity (96%) but poor specificity (31%). These findings are lower than those reported in the initial derivation cohort but broadly consistent with subsequent external validation studies. The score’s modest AUC-ROC (0.73) underlines the need for adjuncts such as imaging in equivocal cases. Post-intervention trends suggest improved adherence to PAS-based risk stratification and reduced avoidable surgeries, although small sample size possibly led to poor statistical power.

Conclusions

Integrating PAS into routine assessment improved risk stratification and reduced the negative appendicectomy rate, although without statistical significance. High scores (≥7) remained highly specific, low scores (≤3) safely ruled out disease, and mid-range scores benefited from adjuncts such as imaging or serial review. Continued use within a structured pathway is advisable, but larger multicentre studies, and the inclusion of imaging adjuncts, are recommended to refine the score and therefore improve diagnostic accuracy and reduce negative appendicectomy rates.

## Introduction

Acute appendicitis is among the most common surgical emergencies in children, yet it remains a diagnostic challenge worldwide in this age group [1]. Untreated appendicitis can lead to serious complications (e.g. perforation and sepsis), whereas overdiagnosing appendicitis can potentially expose patients to avoidable surgical harm. Recent audits have highlighted variability in appendicitis care, emphasising the need for better diagnostic pathways [2].

One response has been the development of clinical risk scores to stratify children by appendicitis probability. The Alvarado score (1986) was an early example used in all ages; many subsequent paediatric tools are variations of this approach [2]. The Paediatric Appendicitis Score (PAS) was specifically introduced by Samuel in 2002 for children, aiming to standardise evaluation of acute abdominal pain [1]. PAS incorporates eight clinical and laboratory variables (total score 0-10) including symptoms (e.g. anorexia, nausea/vomiting), signs (e.g. right iliac fossa tenderness, percussion pain), and inflammatory markers [1]. Higher PAS values reflect greater appendicitis likelihood: scores ≤3 indicate low risk, 4-6 equivocal, and ≥7 high risk of appendicitis [3]. The original prospective study by Samuel of 1,170 children reported a sensitivity of 100% and specificity of 92% for the PAS at a cut-off of ≥6 points, with a positive predictive value (PPV) of 96% and a negative predictive value (NPV) of 99% [1]. Further studies have shown supportive evidence, although not necessarily mirroring the initial derivation. Bhatt et al. reported a specificity of 95.1% at a PAS threshold of ≥8 and a sensitivity of 97.6% at ≤4 in a prospective cohort of children [4]. Similarly, Goldman et al. prospectively validated PAS in 849 children and found an area under the receiver operating characteristic curve (AUC-ROC) of 0.95 [5]. Using cut-offs of ≤2 and ≥7, they reported a 2.4% missed-appendicitis rate and a 4% false-positive rate, supporting PAS as a useful triage aid in highly controlled settings [5].

On the other hand, broader validations have produced more modest results. In the multicentre Right Iliac Fossa Treatment (RIFT) study (2020), 1,827 children across 139 UK and Irish hospitals, the PAS achieved an AUC-ROC of 0.84; at a low-risk cut-off of ≤2, the failure rate was 3.1% and specificity 29.7% [6]. In a separate UK study by Goulder and Simpson, the score was tested retrospectively in 56 surgical patients using the original “operate if ≥6/observe-or-discharge if ≤5” threshold; at that cut-off, PAS showed 87% sensitivity and 59% specificity, meaning 13% of true appendicitis cases would still have been missed [7].

Together, these findings indicate that while PAS retains excellent sensitivity, its stand-alone specificity in unselected real-world cohorts is limited, leading to a higher proportion of false positives. Optimal care still requires complementary clinical judgement and, where available, imaging. Given the diagnostic challenges of paediatric appendicitis and the potential harms of unnecessary surgery, we conducted a two-cycle clinical audit to evaluate the effect of integrating the PAS into routine assessment; the primary objective was to determine whether PAS implementation reduced the negative appendicectomy rate, and the secondary objective was to quantify PAS diagnostic performance against histology at prespecified cut-offs, in line with the 2022 Getting It Right First Time (GIRFT) abdominal pain pathway [2].

## Materials and methods

Study population and design

This retrospective clinical audit was conducted at Southend University Hospital, part of the Mid and South Essex NHS Foundation Trust. The audit followed a two-cycle design. The first audit cycle covered the period from August 2021 to August 2024 and included 156 patients. Data collected included age and gender of the patients, the individual components of the PAS, and the final histological diagnosis. Following the first audit cycle, a focused educational campaign was launched in August 2024: a departmental teaching session introduced a quick-reference guide (QRG) reinforcing routine PAS implementation, in line with the 2022 GIRFT abdominal pain pathway.

The second audit cycle spanned from August 2024 to March 2025 and included 34 patients. The same variables were collected and analysed to assess any change in clinical practice and outcome post-intervention. Data from both cycles were used to assess the impact of the intervention on diagnostic accuracy and surgical decision-making in paediatric appendicitis.

In regards to the sample size, no formal calculation was undertaken. The study size was determined by including all consecutive paediatric appendicectomies performed within the pre-specified audit windows (August 2021-August 2024; August 2024-March 2025). The start date of August 2021 was chosen because the COVID-19-related restrictions on emergency theatre utilisation in our hospital had been lifted by then. Patients were included if they were under 18 years of age and had an appendectomy performed due to clinical suspicion of appendicitis. Exclusion criteria included incomplete clinical records, specifically missing age, PAS component scores or absence of histological outcome data.

Outcome measures

Primary outcome measures were (i) the negative appendicectomy rate in each audit cycle and (ii) the diagnostic performance of the PAS. A negative appendicectomy was defined as histological confirmation of an appendix with no active inflammation after surgical removal. PAS performance was evaluated by calculating sensitivity, specificity, positive predictive value (PPV), negative predictive value (NPV), accuracy, likelihood ratios (LRs), and the AUC-ROC against the histological gold standard.

Intervention

Following the first audit cycle, an educational intervention was implemented in August 2024 to improve adherence to evidence-based assessment of suspected paediatric appendicitis. The intervention began with a formal presentation delivered to the general surgery department. This presentation reviewed the findings from the first audit cycle, with particular focus on the rate of negative appendectomies. Areas for improvement were identified and discussed in collaboration with consultant leads.

Based on the audit findings and departmental consensus, a QRG was developed. This guide summarised key recommendations for clinical decision-making, including the use of the PAS. To support accessibility and routine use, the guide was uploaded to the departmental shared drive and also printed for physical display in key clinical areas: the handover room and the doctors’ office. The intervention aimed to standardise practice, improve the use of risk stratification tools, and reduce the incidence of negative appendectomies.

Statistical analysis

Statistical analysis was performed to evaluate the diagnostic performance of the PAS and changes in clinical practice between the two audit cycles. Descriptive statistics were used to summarise demographic characteristics (age, gender), PAS scores, and histological outcomes. Continuous variables were summarised using means and standard deviations or medians and interquartile ranges (IQRs), while categorical variables were described using frequencies and percentages. All analyses and visualisations were conducted using Python version 3.11.8 (Python Software Foundation, Beaverton, OR, USA), with the pandas, matplotlib, and seaborn libraries. Patients were stratified into three PAS categories based on their total score, following suggested categorisation by Samuel [1]: low-risk: PAS ≤3, equivocal: PAS 4-6, and high-risk: PAS ≥7.

To assess PAS as a diagnostic tool, sensitivity, specificity, PPV, NPV, accuracy, and LRs were calculated using histologically confirmed appendicitis as the reference standard. Diagnostic performance was evaluated at predefined PAS thresholds (≥4, ≥7, ≤3, and ≤2), selected to represent commonly used clinical decision cut-offs [1,5]. ROC analysis was performed with PAS treated as a continuous predictor, and the AUC-ROC was calculated to quantify overall discriminative ability. Comparative statistical testing was planned to evaluate differences in PAS scores between patients with and without histological confirmation of appendicitis, using Welch’s t-test and the Mann-Whitney U test.

Finally, to assess the impact of the educational intervention, pre- and post-intervention comparisons were planned between the two audit cycles. These included comparisons of negative appendectomy rates, PAS distribution, and risk-band frequencies. Differences in proportions were analysed using Chi-squared or Fisher’s exact tests, as appropriate, while differences in continuous variables were evaluated using t-tests or non-parametric equivalents. A p-value of <0.05 was considered statistically significant throughout.

Ethical consideration

This project was conducted as a registered clinical audit within Southend University Hospital, Mid and South Essex NHS Foundation Trust (registration number: GSURG135). All data were collected retrospectively from routine clinical records and were fully anonymised prior to analysis. No identifiable personal information was accessed or stored at any stage of the project, ensuring compliance with data protection and confidentiality standards.

## Results

Descriptive analysis

**Figure 1:**
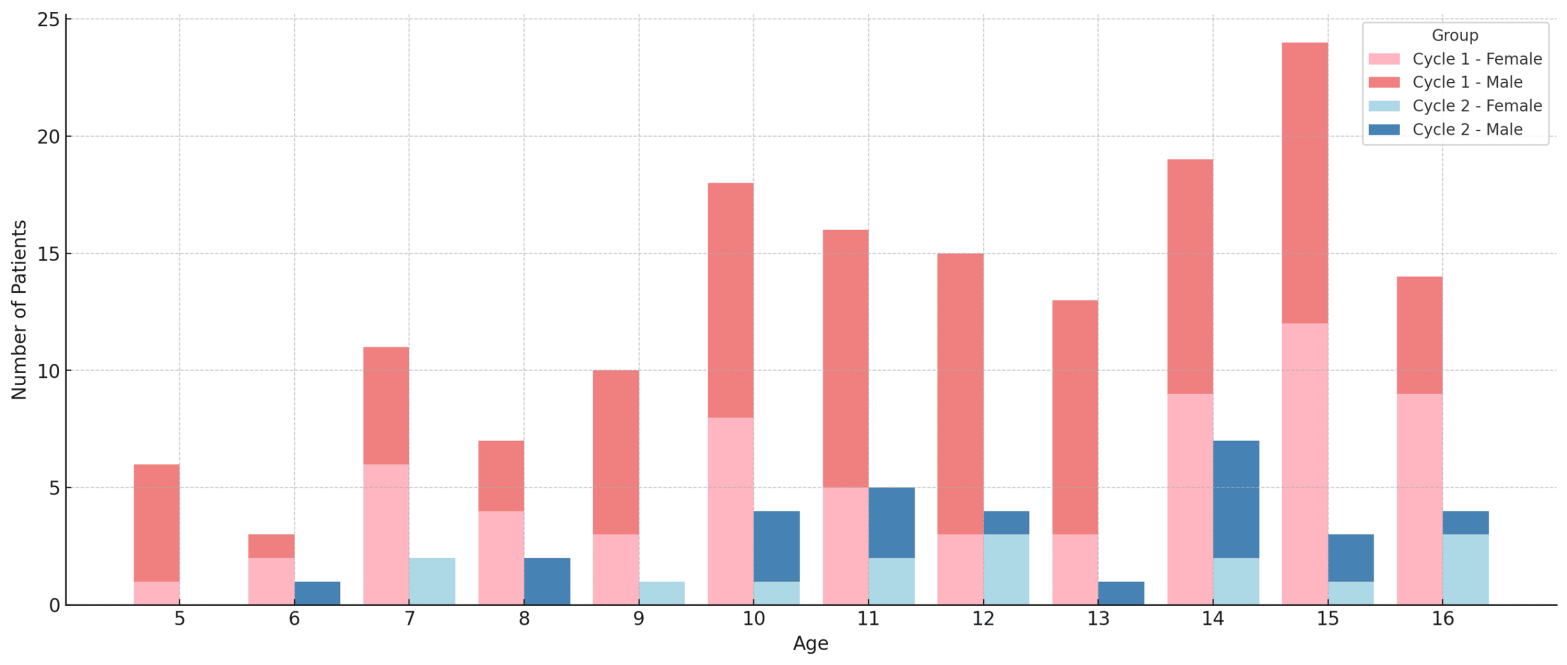
Patient age distribution by audit cycle and gender Stacked bar chart showing the number of operated children at each single year of age, split by audit cycle and sex. Cycle 1 covers August 2021-August 2024 (n=156); Cycle 2 covers August 2024-March 2025 (n=34). The x-axis displays age in years; the y-axis shows the number of patients. Within each age, bars are partitioned into females and males for each cycle, illustrating the age distribution and gender mix across cycles.

A total of 190 paediatric patients who underwent appendectomy were included in the audit, with 156 patients in the first cycle (August 2021-August 2024) and 34 patients in the second cycle (August 2024-March 2025). The mean age across both cycles was approximately 12 years, with a similar IQR (10-14 years). In Cycle 1, 58% of patients were male, compared to 56% in Cycle 2 (Figure 1). The overall histology-confirmed appendicitis rate increased from 71% (110/156) in Cycle 1 to 82% (28/34) in Cycle 2.

**Figure 2:**
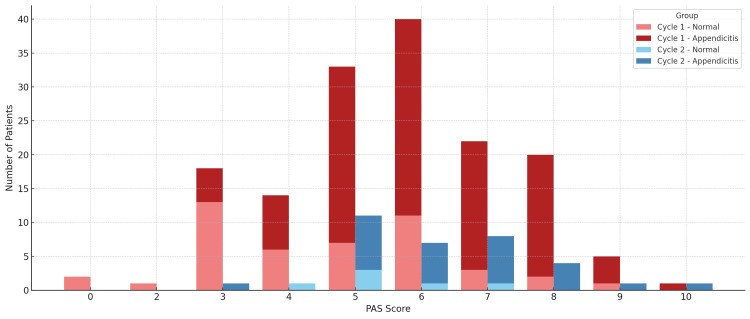
Paediatric Appendicitis Score distribution by audit cycle with histology outcome Stacked bar chart showing the distribution of the Paediatric Appendicitis Score (PAS; 0-10) for operated children in Cycle 1 (August 2021-August 2024; n=156) and Cycle 2 (August 2024-March 2025; n=34). Within each cycle, bars are partitioned by final histology (appendicitis vs normal appendix). The x-axis shows PAS; the y-axis shows the number of patients.

The distribution of PAS scores was comparable between the two cycles, with a median PAS of 6 in both. However, the proportion of patients in the high-risk PAS category (≥7) increased from 31% in Cycle 1 to 41% in Cycle 2, while low-risk cases (PAS≤3) dropped from 13% to just 3%. Intermediate-risk patients (PAS 4-6) remained the largest group in both cycles (Figure 2).

Diagnostic performance of the PAS

**Figure 3:**
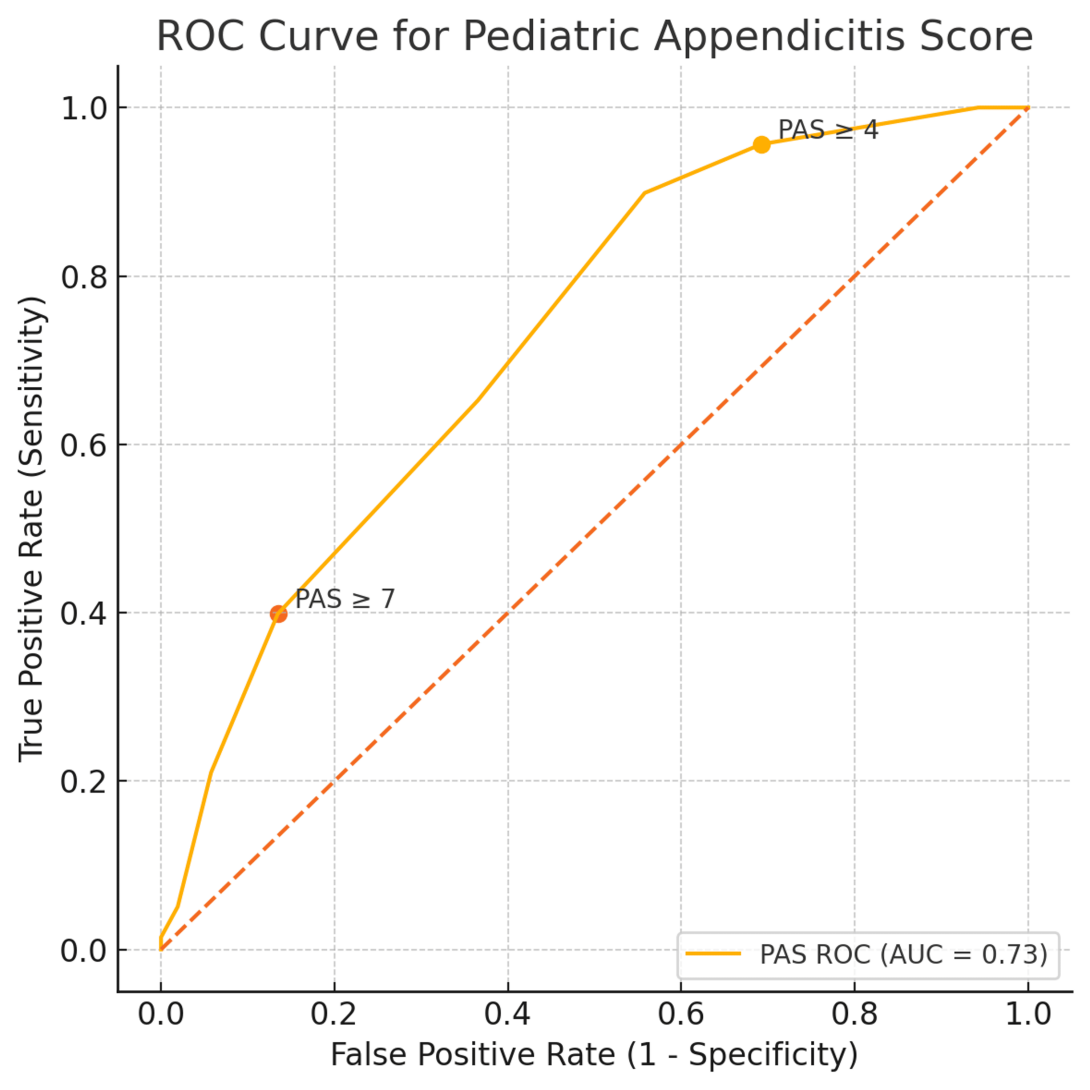
ROC curve for the Paediatric Appendicitis Score (PAS) versus histology Receiver operating characteristic (ROC) curve for PAS (0-10) predicting histology-confirmed appendicitis in the combined operated cohort from Cycle 1 (August 2021-August 2024) and Cycle 2 (August 2024-March 2025) (n=190). The area under the curve (AUC) is 0.73 (95% CI: 0.64-0.81). Axes show sensitivity (y) against false-positive rate=1-specificity (x). The diagonal dashed line denotes chance discrimination. Points are annotated for commonly used thresholds (PAS≥4 and PAS≥7) to illustrate the sensitivity-specificity trade-off. PAS, Paediatric Appendicitis Score; ROC, receiver-operating-characteristic; AUC, area under the curve; CI, confidence interval.

Table 1 and Figure 3 summarise the discriminatory ability of the PAS in the combined cohort (n=190). At the threshold of ≥4, PAS detected 96% (95% CI: 91%-98%) of histology-confirmed cases but achieved limited specificity of 31% (95% CI: 20%-44%). The resultant PPV was 0.79 (95% CI: 0.72-0.84) and the NPV 0.73 (95% CI: 0.52-0.87), yielding an overall accuracy of 0.78.

**Table 1:** Diagnostic accuracy of the Paediatric Appendicitis Score at pre-specified decision thresholds (95% confidence intervals in parentheses) Data include all paediatric appendicectomies from Cycle 1 (August 2021-August 2024; n=156) and Cycle 2 (August 2024-March 2025; n=34). Histology was the reference standard. For rule-in thresholds (PAS≥4, ≥7), a positive test is PAS≥cut-off. *For rule-out thresholds (PAS≤3, ≤2), a negative test is PAS≤cut-off, and TP/FP/FN/TN are re-labelled accordingly. Proportions are shown with 95% CIs. PAS, Paediatric Appendicitis Score; TP, true positives; FP, false positives; FN, false negatives; TN, true negatives; PPV, positive predictive value; NPV, negative predictive value; LR+, positive likelihood ratio; LR-, negative likelihood ratio; CI, confidence interval.

Metric/Count (95% CI)	PAS≥4	PAS≥7	PAS≤3 *	PAS≤2 *
TP	132	55	132	138
FP	36	7	36	49
FN	6	83	6	0
TN	16	45	16	3
Sensitivity	0.96 (0.91-0.98)	0.40 (0.32-0.48)	0.96 (0.91-0.98)	1.00 (0.97-1.00)
Specificity	0.31 (0.20-0.44)	0.87 (0.75-0.93)	0.31 (0.20-0.44)	0.06 (0.02-0.16)
PPV	0.79 (0.72-0.84)	0.89 (0.78-0.94)	0.79 (0.72-0.84)	0.74 (0.67-0.80)
NPV	0.73 (0.52-0.87)	0.35 (0.27-0.44)	0.73 (0.52-0.87)	1.00 (0.44-1.00)
Accuracy	0.78	0.53	0.78	0.74
LR+	1.38	2.96	1.38	1.06
LR-	0.14	0.70	0.14	0.00

Raising the cut-off to ≥7 improved specificity to 87% (95% CI: 75%-93%) and produced a high PPV of 0.89 (95% CI: 0.78-0.94). However, sensitivity fell to 40% (95% CI: 32%-48%), with a corresponding LR+ of 3.0 and LR- of 0.70. When used as a negative test with a cut-off of ≤3, the PAS preserved high sensitivity at 96% (95% CI: 91%-98%) with an LR- of 0.14. Only 31% of patients without appendicitis fell below this cut-off, and the NPV was 0.73 (95% CI: 0.52-0.87). Reducing the boundary further to ≤2 eliminated false negatives altogether, but applied to just three children and reduced specificity to 6% (95% CI: 2%-16%). The AUC-ROC was 0.73 (95% CI: 0.64-0.81). Score frequencies were ≤3 in 21/190 (11%), 4-6 in 112/190 (59%), and ≥7 in 57/190 (30%).

Comparison of PAS between histology groups

**Figure 4:**
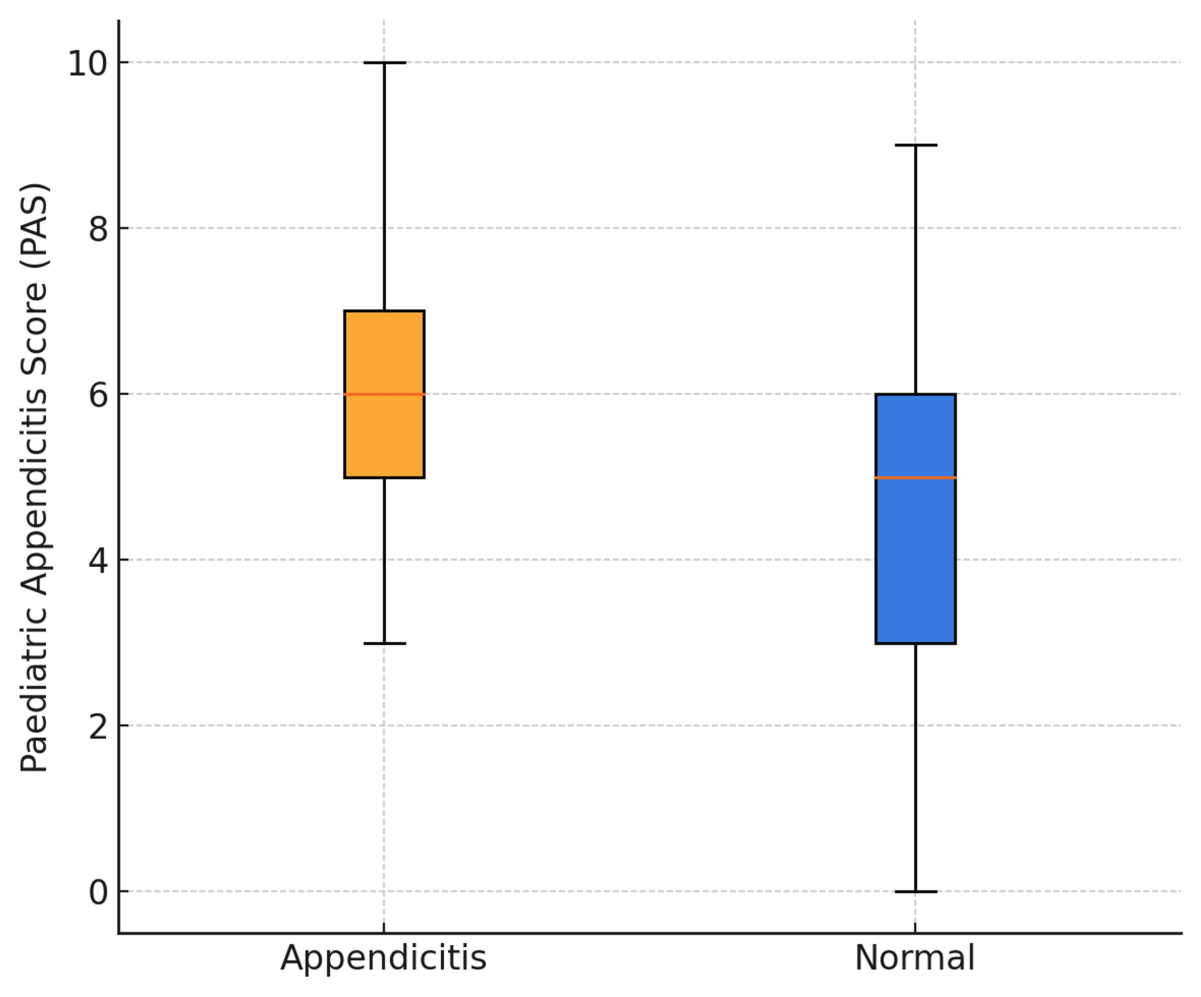
Distribution of Paediatric Appendicitis Scores (PAS) by histology outcome Boxplots of PAS (0-10) for children with histology-confirmed appendicitis (n=138) versus a normal appendix (n=52). Boxes show the interquartile range (IQR) with median line; whiskers extend to 1.5×IQR. Mean PAS was 6.18±1.50 vs 4.69±1.85; mean difference 1.49 (95% CI: 0.92-2.06). PAS, Paediatric Appendicitis Score; IQR, interquartile range; CI, confidence interval.

Children with histology-confirmed appendicitis (n=138) had a higher mean PAS than those with a normal appendix (n=52): 6.18±1.50 vs 4.69±1.85, giving a mean difference of 1.49 points (95% CI: 0.92-2.06). Welch’s unequal-variance t-test showed the difference was significant (t=5.19, p=1.6 × 10^-^⁶). The non-parametric Mann-Whitney U test confirmed this finding (U=5.214, p=9.9 × 10^-^⁷). Medians followed the same pattern (6 (IQR: 5-7) vs 5 (3-6)) (Figure 4).

Comparison of pre- and post-intervention cycles

Cycle 1 included 156 children, whereas Cycle 2 comprised 34.

Negative Appendectomy Rate

Forty-six negative appendectomies occurred in Cycle 1 (29.5%) compared with six in Cycle 2 (17.6%). The reduction of 11.9 percentage points was not statistically significant (χ²=1.97, p=0.16).

PAS Distribution

Mean PAS rose slightly after the educational intervention (5.68±1.77 vs 6.21±1.47). A Welch unequal-variance t-test did not detect a significant difference (t=-1.82, p=0.075). Medians were identical at 6 (IQR: 5-7) in both cycles (Mann-Whitney U=2.262, p=0.17).

Risk-Band Frequencies

Low-risk scores (≤3) fell from 21/156 (13%) to 1/34 (3%), intermediate scores (4-6) were 87/156 (56%) versus 19/34 (56%), and high-risk scores (≥7) increased from 48/156 (31%) to 14/34 (41%). The overall shift in distribution was not significant (χ²=3.59, p=0.17).

## Discussion

Over a three-year baseline period followed by a six-month post-intervention phase, we audited 190 paediatric appendicectomies (156 pre-intervention, 34 post-intervention) to evaluate the routine use of the PAS in a UK district-general hospital. The mean patient age was about 12 years with an IQR of 10-14, matching the demographics in the derivation cohort by Samuel and in the multicentre RIFT audit [1,6]. Male predominance remained stable (58% vs 56%), again mirroring the 55%-65% reported elsewhere [1,6].

At the high-risk cut-off of PAS ≥7, our audit achieved 87% specificity (PPV=0.89) but only 40% sensitivity, highlighting the familiar rule-in trade-off (high specificity with low sensitivity). The derivation cohort by Samuel is the notable exception: by setting the PAS threshold at ≥6, he reported 100% sensitivity alongside 92% specificity [1]. In later validation cohorts, sensitivity declines at higher thresholds: Goldman et al. observed 61% sensitivity with 96% specificity at ≥7, while Bhatt et al. recorded 42% sensitivity and 95% specificity at ≥8 [4,5]. In these two studies, in addition to our own, sensitivity drops as the threshold rises: while high PAS values confirm appendicitis reliably, they still miss some cases. This variation shows that thresholds optimised in one cohort often perform less well elsewhere, underscoring the need for external validation. Conversely, using PAS ≥4 to maximise sensitivity kept missed cases low (96% sensitivity) but specificity collapsed to 31%, almost identical to 33% reported by Bhatt et al. at the same cut-point; this confirms that low thresholds are useful for ruling out disease rather than ruling it in [4]. Likewise, Kharbanda et al. validated a three-item “Low-Risk Appendicitis Rule”: absence of right lower quadrant maximal tenderness, no nausea, and an absolute neutrophil count ≤6.75 × 10⁹ L⁻¹ [8]. This achieved 95.5% sensitivity but only 36% specificity in a multicentre cohort of 2,625 children, highlighting the same high-sensitivity/low-specificity trade-off [8].

Within the “rule-out” band, PAS ≤3 gave an NPV of 0.73 with a ~4% missed appendicitis rate, whereas tightening to ≤2 pushed the NPV to 1.00 and eliminated missed cases. This mirrored the report by Goldman et al., who found only 2.4% misses at this ultra-low score [5]. The overall AUC-ROC of 0.73 denotes moderate discriminative power, lower than the 0.84 seen in the multicentre RIFT audit and well below the 0.95 reported by Goldman et al., reflecting the variability inherent in real-world practice [5,6]. A 2013 systematic review of six paediatric appendicitis rules (PAS, Alvarado score, Kharbanda low-risk rule, Lintula score, Modified Lindberg score, and the van den Broek rule) by Kulik et al. reached the same conclusion, noting that although PAS and the Alvarado score were the most extensively validated tools, neither consistently met prespecified accuracy benchmarks across cohorts [9].

Mean PAS was nearly 1.5 points higher in children with histology-proven appendicitis than in those with a normal appendix (6.18±1.50 vs 4.69±1.85), a difference that remained highly significant on both parametric and non-parametric testing. A similar ~1.5-point difference was documented in the derivation cohort by Samuel and in the validations by Goldman et al. and Bhatt et al. [1,4,5]. However, the IQRs still overlap (5-7 vs 3-6). This overlap restricts discrimination (AUC-ROC=0.73) and underscores the need for additional predictors (e.g. imaging or serial review) in equivocal cases. Hence, although the mean difference supports PAS as a useful triage aid, it cannot replace experienced clinical judgement and additional diagnostics.

Following the educational intervention, the negative-appendicectomy rate fell from 29.5% to 17.6%, a clinically notable 12-point drop that did not reach statistical significance. This was an unsurprising result given the limited post-intervention sample (n=34). Mean PAS rose modestly (from 5.68 to 6.21) with the median unchanged at 6, while the distribution shifted: low-risk scores (≤3) nearly disappeared (from 13% to 3%) and high-risk scores (≥7) increased from 31% to 41%. Although these changes were also non-significant, the pattern indicates better adherence to the scoring protocol and more selective surgery; a larger or longer series will be needed to confirm statistical impact.

Strengths

This audit used a before-and-after design in a real-world district-general hospital, allowing direct assessment of practice change over time. Histology served as the reference standard, minimising diagnostic misclassification, and every paediatric appendicectomy over three years was included, giving a representative baseline. The post-intervention phase applied a defined educational campaign, demonstrating that even a modest intervention can shift score distribution and numerically lower the negative-appendicectomy rate.

Limitations

Findings come from a single centre and the post-intervention cohort were small (n=34), restricting statistical power and preventing firm causal inference. Baseline PAS values were retrospectively taken from notes, introducing potential documentation bias, and the study did not capture imaging utilisation. The six-month follow-up may also be too short to account for sustained change in practice.

We recommend continuing routine PAS use but pairing intermediate scores with additional predictors (e.g. timely imaging or serial examination) to increase specificity. This approach mirrors the work of Shah et al., whose PAS-based pathway cut CT use from 75% to 24% while maintaining a pathway sensitivity of 98%-99%, emphasising the advantages of structured scoring-plus-imaging algorithms [10]. Future work should analyse the individual PAS components to determine which clinical or laboratory variables offer the greatest discriminatory value, which could allow less informative items to be modified or omitted. Expanding the audit across multiple centres and over a longer timeframe will provide the statistical power needed for definitive conclusions and enhance generalisability. Finally, ongoing refresher training, together with exploration of other decision tools such as the Paediatric Appendicitis Risk Calculator (pARC) [11], systematic evidence clarifying the most clinically useful thresholds for Alvarado and PAS [12], and emerging biomarkers [13], could further refine risk stratification and minimise avoidable surgery.

## Conclusions

In summary, integrating the PAS into everyday practice at our district-general hospital improved risk stratification and lowered the negative-appendicectomy rate, although without statistical significance in this limited post-intervention sample. High PAS thresholds (≥7) proved reliably specific, while very low scores (≤2-3) offered safe rule-out capability. Nonetheless, intermediate scores still require the use of additional predictors (e.g. imaging or serial review). These findings support continued PAS use within a structured pathway and highlight the need for larger, multicentre studies to confirm sustained benefits and to refine the score’s components for even greater diagnostic accuracy.
